# A Start Codon Variant in* NOG* Underlies Symphalangism and Ossicular Chain Malformations Affecting Both the Incus and the Stapes

**DOI:** 10.1155/2019/2836263

**Published:** 2019-07-22

**Authors:** Nathan R. Lindquist, Eric N. Appelbaum, Anushree Acharya, Jeffrey T. Vrabec, Suzanne M. Leal, Isabelle Schrauwen

**Affiliations:** ^1^Department of Otolaryngology, Head and Neck Surgery, Baylor College of Medicine, Houston, Texas, USA; ^2^Center for Statistical Genetics, Department of Neurology, Gertrude H. Sergievsky Center, Columbia University Medical Center, New York, NY, USA; ^3^Department of Otolaryngology, Houston Methodist Hospital, Houston, Texas, USA

## Abstract

We performed exome sequencing to evaluate the underlying molecular cause of a patient with bilateral conductive hearing loss due to multiple ossicular abnormalities as well as symphalangism of the fifth digits. This leads to the identification of a novel heterozygous start codon variant in the* NOG* gene (c.2T>C:p.Met1?) that hinders normal translation of the noggin protein. Variants in* NOG* lead to a spectrum of otologic, digit, and joint abnormalities, a combination suggested to be referred to as* NOG*‐related‐symphalangism spectrum disorder (*NOG*‐SSD). Conductive hearing loss from such variants may stem from stapes footplate ankylosis, fixation of the malleoincudal joint, or fixation of the incus short process. In this case, the constellation of both stapes and incus fixation, an exceptionally tall stapes suprastructure, thickened long process of the incus, and enlarged incus body was encountered, leading to distinct challenges during otologic surgery to improve hearing thresholds. This case highlights multiple abnormalities to the ossicular chain in a patient with a start codon variant in* NOG*. We provide detailed imaging data on these malformations as well as surgical considerations and outcomes.

## 1. Introduction

The* NOG* gene encodes for the secreted protein noggin, which is crucial in normal bone and joint development. Noggin antagonizes bone morphogenetic proteins (BMPs) signaling, which are multifunctional growth factors that belong to the transforming growth factor beta (TGFbeta) superfamily and are important in the development of various tissues including bone and cartilage [1].

Autosomal dominantly inherited or* de novo* variants in* NOG* lead to a spectrum of joint/digit abnormality disorders with or without conductive hearing loss, including Type B2 brachydactyly (BDB2), multiple synostoses syndrome 1 (SYN1), stapes ankylosis with broad thumbs and toes (Teunissen–Cremers syndrome), proximal symphalangism 1A (SYM1A), and tarsal-carpal coalition syndrome (TCC). Overall, this can be referred to as* NOG*‐related‐symphalangism spectrum disorder (*NOG*‐SSD) in a single unified diagnostic term [2]. The conductive hearing loss is usually due to ankylosis of the stapes. Other abnormalities of the ossicular chain can also occur but are less frequent [2]. Heterozygous Nog+/− mice display mild conductive hearing loss due to stapes fixation [3].

Herein, we describe a novel variant in the* NOG* gene causing NOG symphalangism syndrome (NOG-SSD) including incus malformations and report its clinical and molecular phenotype as well as surgical considerations and results for the patient's conductive hearing loss.

## 2. Materials and Methods

### 2.1. Patient

After IRB approval (H-17566) at the Baylor College of Medicine, the patient's complete medical and family history was reviewed with attention to symphalangism and conductive hearing loss. Study participants provided written, informed consent. Pre- and postoperative audiometry included pure tone audiometry and tympanometry, as well as ipsilateral and contralateral stapedial reflex testing. High-resolution computed tomography (CT) of the temporal bone without contrast was performed utilizing axial slices with coronal and sagittal reconstructions to evaluate the middle and inner ears.

### 2.2. Exome Sequencing

A saliva sample was obtained from the patient using the Oragene OG-500 collection kit and DNA was extracted via the prepIT purification kit (DNA Genotek, Ontario, Canada). Exomic libraries were prepared using the SureSelect Human All Exon V6 kit, and sequencing was performed on a HiSeq (2500/4000) instrument. Reads were aligned to the human reference genome using Burrows-Wheeler Aligner (BWAv0.7.15) [4]. Local realignment around Insertion/Deletions (InDels), base quality score recalibration, duplicate reads removal, and single nucleotide and InDel variant calling were performed by the Genome Analysis Toolkit (GATKv3.7) and Picard tools [5]. Next, annotation was performed using ANNOVAR (v2018Apr16), and variants were filtered based on frequency in databases [Genome Aggregation Database (gnomAD) minor allele frequency <0.005] [6]. Various bioinformatic predictions were used to evaluate variants (obtained from dbnsfp33a and ANNOVAR) [7]. Sanger sequencing was performed to verify a variant identified in* NOG*. In short, purified PCR products (Primers: Forward: 5′-GCCAACTTGTGTGCCTTTCT-3′; Reverse 5′-GTCTGGGTGTTCGATGAGGT-3′) were sequenced using the BigDye Terminator v3.1 Cycle Sequencing Kit followed by capillary electrophoresis on an ABI 3730 DNA Analyzer (Applied Biosystems Inc., Foster City, CA).

## 3. Results

### 3.1. Clinical Features

**Figure 1:**
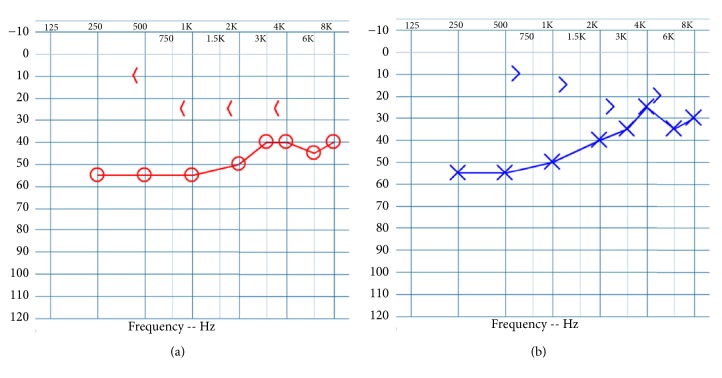
Preoperative pure tone audiometry revealed bilateral moderate rising to mild conductive hearing loss on the right (a) and left (b). Ipsilateral and contralateral stapedial reflexes were absent bilaterally.

**Figure 2:**
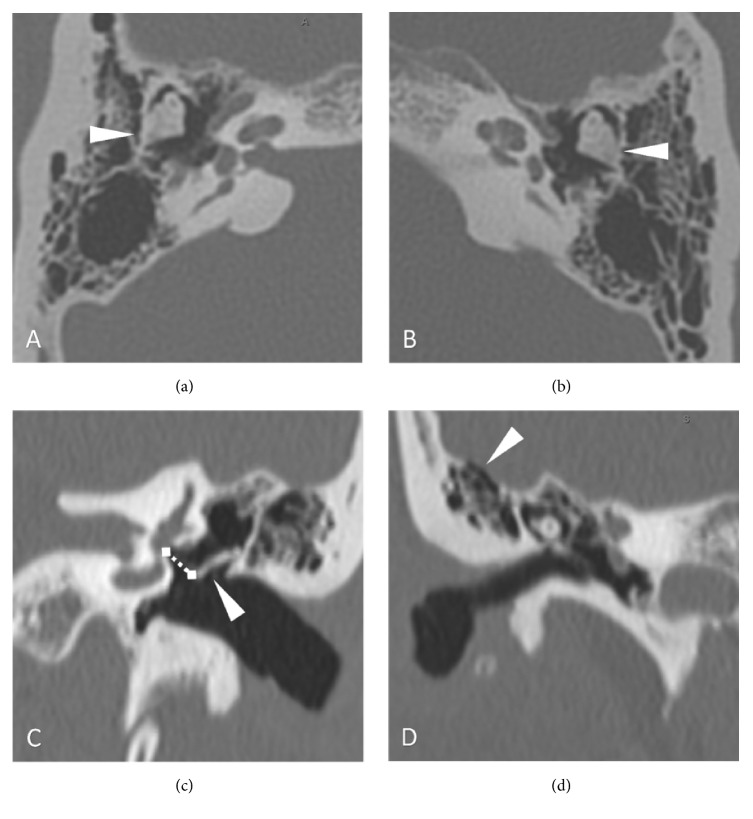
Axial CT bone windows of the temporal bones revealed a markedly enlarged incus with possible fixation in both the right (a) and the left ears (a). Coronal reformats on the left (c) demonstrated an elongated stapes superstructure of approximately 4.8 mm (dashed line) with thickening of the incus long process (arrowhead). On the right (d), this measurement was 4.5 mm. Coronal views of the right ear demonstrated bony dehiscence or thinning in the tegmen mastoideum without meningoencephalic herniation. Similar findings were noted on the left.

Our patient is a 35-year-old Caucasian male referred for bilateral progressive conductive hearing loss since early childhood. Evaluation at the age of ten included audiometry but no imaging. Reportedly, this demonstrated some degree of hearing loss but was not available for review. Medical history included an abdominal lipoma, open heart surgery for aortic valve anomaly at the age of 5, and multiple orthopedic surgeries including excision of a ganglion cyst of the wrist, as well as repair of shoulder and ankle fractures from injury-appropriate sports-related trauma not thought to be related to his phenotype. Notably, the patient denied ophthalmologic history. He had a family history of hearing loss in his father and paternal grandfather. Reportedly, his grandfather had similar symphalangism of both fifth digits, while his father did not. Neither were available for genetic testing or examination. Physical examination of the hands demonstrated symphalangism of both fifth digits only. He did not have proximal symphalangism of the other digits or noted brachydactyly. Imaging of the hands was not performed at our institution due to prior work-up with his primary physicians. Binocular microscopy demonstrated bilateral normal tympanic membranes and well-aerated middle ear spaces, and pneumatic otoscopy displayed mobility of the malleus bilaterally. Pure tone audiometry revealed bilateral moderate rising to mild conductive hearing loss bilaterally (Figure 1). Speech recognition threshold was 45 dB in the right ear and 40 dB in the left, with word recognition scores for single word understanding in quiet of 90% and 100% at 70 dB HL, respectively. Acoustic immittance results revealed normal eardrum mobility, and ipsilateral and contralateral stapedial reflexes were absent bilaterally. CT imaging of the temporal bones revealed an enlarged incus with possible fixation in both the right (Figure 2(a)) and the left (Figure 2(b)) ears. Coronal reformats demonstrated an elongated stapes superstructure of approximately 4.5 mm on the right and 4.8 mm on the left with thickening of the incus long process bilaterally (Figure 2(c)). Tegmen dehiscence or thinning was noted bilaterally in the tegmen mastoideum without meningoencephalic herniation (Figure 2(d)).

### 3.2. Surgical Interventions

**Figure 3:**
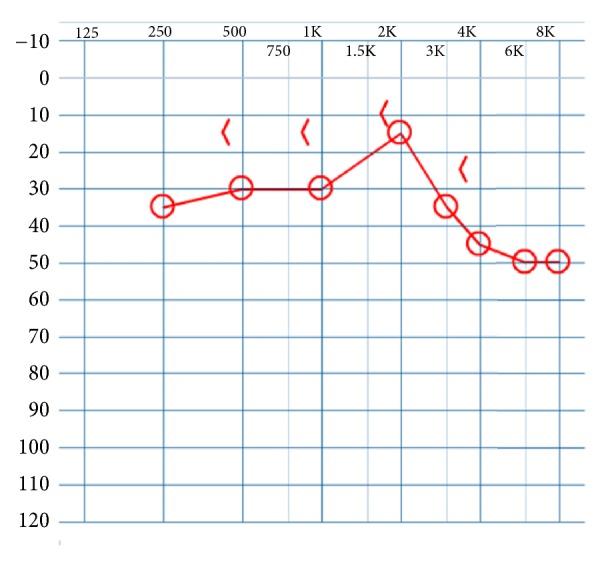
Postoperative pure tone audiometry revealed improvement of the air conduction thresholds to a mild conductive hearing loss with near closure of the air-bone gap on the right. Unfortunately, the patient has not followed up for postoperative audiogram after his second operation but reports subjectively improved and symmetric hearing.

After appropriate counselling, the patient underwent surgical management to address his right conductive hearing loss. Intraoperatively, ossicular abnormalities including an enlarged, fixed incus as well as fixed stapes were confirmed. The incus was mildly fixed and was mobilized using gentle traction between the body and the scutum. The patient underwent right stapedotomy with CO2 laser and reconstruction with a 5 mm Richards piston over fascia seal of the oval window. His postoperative course was unremarkable. The patient's postoperative pure tone and speech recognition thresholds improved significantly after surgery (Figure 3). He underwent interval surgery for the left ear five months later. Intraoperative findings were identical and included exceptionally tall stapes and thickened long process of the incus. Initially, a Robinson prosthesis was selected; however, it would not seat properly on the enlarged lenticular process. Therefore, a 5.5 mm Causse piston was selected for reconstruction with good results. Unfortunately, the patient has not obtained a postoperative audiogram after his second surgery but does report improved, symmetric subjective hearing bilaterally at his most recent clinical follow-up.

### 3.3. Genetic Analysis

Exome sequencing revealed a heterozygous start codon variant in the* NOG *gene in the patient (NM_005450: c.2T>C:p.Met1?), which was verified by Sanger sequencing. This variant is absent from the gnomAD database and is predicted to be deleterious by various bioinformatic tools included in dbnsfp33a (e.g., VariantTaster, FATHMM, SIFT) [7]. The variant leads to a loss of the initiation codon (Methionine), hinders translation of the wild-type Noggin protein, and was classified as likely pathogenic (according to the American College of Medical Genetics (ACMG) guidelines for the interpretation of sequence variants) [8].

## 4. Discussion

In patients with* NOG *variants, conductive hearing loss has mainly been described as a result from stapes ankylosis. However, other ossicular chain abnormalities have also been observed in a few cases, such as fixation of the malleoincudal joint, fixation of the short process of the incus, and elongation of the long process of the incus [2]. Preoperative imaging for this case demonstrated a tall stapes superstructure (increased distance from the incus to the vestibule), thickened long process of the incus, and enlarged incus body. Intraoperatively, the stapes and incus were both noted to be fixed. After the mobilization of the incus, stapedotomy, and fascial graft placement at the oval window, the main intraoperative challenge was selection of the appropriate stapes prosthesis given these anatomic abnormalities. The thick incus long process makes placement of clip type prosthesis difficult and renders standard crimping tools less useful. Bucket-handle prostheses are also difficult given the girth of the incus lenticular process. The CT findings in our case show a very large incus, not illustrated in other cases of* NOG* variants. Thus, we feel CT imaging is useful in preoperative planning to anticipate difficulties with prosthesis placement.

Variable hearing results are reported after stapedectomy for* NOG* variants [9–11]. Brown et al. noted a high prevalence of bone regrowth over the footplate resulting in recurrent conductive loss [9]. All authors recognize the need for prosthesis that is longer than average. Temporal bone histopathology does not demonstrate inner ear anomalies [8].

In conclusion, exome sequencing was utilized to identify a novel variant in the* NOG *gene (c.2T>C:p.Met1?) in a patient with bilateral progressive conductive hearing and symphalangism in the fifth digits. This variant leads to a loss of the translation start site of the* NOG *gene and is predicted to lead to a complete loss of function of the noggin protein. We highlight that multiple malformations in the middle ear ossicular chain can exist with* NOG* variants.

## References

[1] Chen D., Zhao M., Mundy G. R. (2004). Bone morphogenetic proteins. *Growth Factors*.

[2] Potti T. A., Petty E. M., Lesperance M. M. (2011). A comprehensive review of reported heritable noggin-associated syndromes and proposed clinical utility of one broadly inclusive diagnostic term: NOG-related-symphalangism spectrum disorder (NOG-SSD). *Human Mutation*.

[3] Hwang C. H., Wu D. K. (2008). Noggin heterozygous mice: an animal model for congenital conductive hearing loss in humans. *Human Molecular Genetics*.

[4] Li H., Durbin R. (2010). Fast and accurate long-read alignment with Burrows-Wheeler transform. *Bioinformatics*.

[5] McKenna A., Hanna M., Banks E. (2010). The genome analysis toolkit: a MapReduce framework for analyzing next-generation DNA sequencing data. *Genome Research*.

[6] Wang K., Li M., Hakonarson H. (2010). ANNOVAR: functional annotation of genetic variants from high-throughput sequencing data. *Nucleic Acids Research*.

[7] Liu X., Wu C., Li C., Boerwinkle E. (2016). dbNSFP v3.0: a one-stop database of functional predictions and annotations for human nonsynonymous and splice-site SNVs. *Human Mutation*.

[8] Quesnel A. M., Nadol J. B., Nielsen G. P., Curtin H. D., Lesperance M. M. (2015). Temporal bone histopathology in NOG-symphalangism spectrum disorder. *Otology & Neurotology*.

[9] Brown D. J., Kim T. B., Petty E. M. (2003). Characterization of a stapes ankylosis family with a nog mutation. *Otology & Neurotology*.

[10] Coombs A. C., Bird P. A. (2016). Stapedectomy in teunissen–cremers syndrome. *Otology & Neurotology*.

[11] Weekamp H. H., Kremer H., Hoefsloot L. H. (2005). Teunissen-Cremers syndrome: a clinical, surgical, and genetic report. *Otology & Neurotology*.

